# Comprehensive genome analysis of *Streptomyces caeruleatus* S14 isolated from rice rhizosphere

**DOI:** 10.3389/fpls.2025.1526700

**Published:** 2025-03-26

**Authors:** Sucharita Mohapatra, S. R. Prabhukarthikeyan, Gayatri Biswal, Mihira Kumara Mishra, Soumya Shephalika Dash, Gyanisha Nayak, U. Keerthana, C. Parameswaran, P. Panneerselvam, S. D. Mohapatra

**Affiliations:** ^1^ ICAR-National Rice Research Institute, Cuttack, India; ^2^ Department of Plant Pathology, College of Agriculture, Odisha University of Agriculture and Technology, Bhubaneswar, India

**Keywords:** actinobacteria, biocontrol, genome annotation, genome sequence, rice blast, *Streptomyces caeruleatus*, whole genome sequence

## Abstract

Rice blast, caused by *Magnaporthe oryzae*, is one of the most devastating diseases affecting rice crops. We investigated effectiveness of *Streptomyces* spp. against *M. oryzae*. The results revealed that among the *Streptomyces* spp., *Streptomyces caeruleatus* strain S14 demonstrated superior effectiveness in inhibiting the mycelial growth of *M. oryzae* (74.7%). The strain was identified by sequencing 16S rRNA region. Further, the complete genome sequence of this highly effective strain was acquired using the Illumina NovaSeq 6000 (PE 150), revealing a total genome length of 9,750,804 base pairs (9.7 Mb). The genome comprises 9,191 protein-coding sequences (CDS), 68 transfer RNA (tRNA) genes, 6 ribosomal RNA (rRNA) genes, with an average G+C content of 71.03%. The *Streptomyces caeruleatus* S14 genome, annotated with RASTtk and genetic code 11, falls under the superkingdom Bacteria. According to annotation statistics from PATRIC, it is a high-quality genome with 97.9% coarse consistency, 93.7% fine consistency, and completeness of 99.9%. The genome included genes related to metabolism, protein processing, defense, virulence, energy, stress response, membrane transport, regulation, cell signaling, cell envelope, DNA processing, cellular activities, RNA processing, and miscellaneous. The complete genome sequence of *S. caeruleatus* suggests that it offers valuable insights into its antimicrobial activity and provide key genetic traits responsible for pathogen suppression. Incidentally this is the first whole genome sequencing report of *S. caeruleatus* isolated from rice rhizosphere soil in India.

## Introduction

Rice (*Oryza sativa* L.) is a vital food crop and a staple diet for people worldwide, particularly in Asia. However, its production is affected by various biotic and abiotic stresses (Chaiharn et al., 2009). Rice blast, caused by the filamentous, ascomycetous fungus *M. oryzae*, is a major biotic stress for rice crops. The yield losses from rice blast vary between 10-30% in different rice-producing countries, with losses potentially reaching up to 50% during severe disease outbreaks (Asibi et al., 2019; Luh Suriani et al., 2020). To increase agricultural production, modern agriculture often depends on fungicides. However, the usage of fungicides result in environmental pollution, pesticide resistance, residual issues, soil quality degradation, and damages the natural ecosystems (Pooja and Katoch, 2014; Law et al., 2017). To mitigate the detrimental effects of these toxic chemicals, biocontrol agents could be regarded as essential candidates for promoting sustainable agriculture (Kenawy et al., 2019; Kalam et al., 2020; Basu et al., 2021; Naz et al., 2022; Vinothini et al., 2024). Biocontrol of plant diseases is a time-consuming process with little immediate profits, but it offers long-lasting, cost-effective, and safe solutions for managing plant health (Prabhukarthikeyan et al., 2019; Sawant et al., 2023, 2024). Nowadays biological control by using potential actinomycetes is receiving greater attention all over the world. Many of them belong to the group of Plant growth-promoting rhizobacteria (PGPR) (Jones and Elliot, 2017).

Actinobacteria, commonly known as actinomycetes is a phylum of Gram-positive, filamentous bacteria with a high % G+C content and they are found in larger range of habitats, which include both terrestrial and aquatic ecosystems (Amin et al., 2019; Rego et al., 2019; Omar et al., 2022). Because of its effective secondary metabolite synthesis, this phylum is important in the sectors of agriculture, medicine, and industry (Harir et al., 2018; Ferraiuolo et al., 2021). Actinobacteria have attracted a lot of attention from the scientific community for their ability to produce a wide range of bioactive compounds with antimicrobial, antitumoral, and immunosuppressant activities, making them a key focus of research (Barka et al., 2016; Sudha et al., 2022). Actinobacteria play a crucial role in suppressing fungal pathogens in both controlled environments and living organisms by triggering key genes involved in systemic acquired resistance (SAR) and the jasmonate/ethylene (JA/ET) pathways (Conn et al., 2008). They employ diverse antagonistic strategies, including competition for space and nutrients, antibiotic production, siderophore secretion, lytic enzyme release, volatile organic compound (VOC) emission and stimulation of host resistance (Gong et al., 2022; Kaari et al., 2022).

*Streptomyces* are known to be the largest genus of phylum Actinobacteria, which are ubiquitous in soil. They are essential in the biological buffering of soils and are integral to the decomposition of organic matter, which is essential for promoting crop production. The production of antifungal compounds and extracellular hydrolytic enzymes by various *Streptomyces* spp. has been extensively studied, highlighting their significant role in biocontrol, particularly against phytopathogens including rice blast disease (Gonzalez-Franco and Robles-Hernandez, 2009; Dave et al., 2024). For this reason, the isolation, characterization and identification of novel and efficient *Streptomyces* strains from natural resources is an important achievement. Bacterial genomes can be examined using whole genome sequencing (WGS), which is essential for genome annotation, revealing numerous genes with homology to known transporters, drug targets, virulence factors, and antibiotic resistance genes. *Streptomyces* spp. are known to have a linear chromosome ranging in size from 5.1 to 10.1 Mbp (Kirby et al., 2012; Lee et al., 2020). There are many different types of sequencing have been used for Whole genome sequencing. WGS of *Streptomyces* bacteria has been done using both PacBio and Illumina. PacBio provides long reads and relatively expensive, while Illumina is cost-effective and produces short reads. But by far the most practical microbial genomics sequencing platform is Illumina (Shin et al., 2013; Rhoads and Au, 2015).

In this study, we reported the complete genome sequence of *S. caeruleatus*, which was isolated from rhizosphere soil of rice from Cuttack district, Odisha, India. To the best of our knowledge, this is the first WGS report of *S. caeruleatus* which was isolated from rice rhizosphere soil in India.

## Materials and methods

### Bacterial isolates

*Streptomyces* spp. were isolated from rice rhizosphere soil of Odisha, India (**Supplementary Table S1**). Soil sample was serially diluted and spread evenly over the surface of SCA (Starch Casein Agar) media treated with nystatin (50 μg/ml) and rifampicin (2.5 μg/ml) in a Petridish to prevent fungal growth. After seven days of incubation at 30°C, the morphologically different *Streptomyces* colonies were collected and inoculated into International *Streptomyces* Project (ISP2) agar medium, which was then incubated for seven days at 28°C (Shirling and Gottlieb, 1966). Then the *Streptomyces* isolates were stored at -20°C in glycerol stocks for further use.

### Pathogen

*M. oryzae* isolate RLB 06 (NCBI accession number- MT093385) was obtained from Plant pathology, Division of Crop protection, ICAR- National Rice Research Institute, Cuttack, Odisha, India.

### Antifungal activity of *Streptomyces* spp. against *Magnaporthe oryzae*

The dual culture method was used to assess the antifungal activity of *Streptomyces* spp. against *M. oryzae* (Khare et al., 2010). *Streptomyces* isolates were cultured on ISP2 medium for seven days, and a loopful of each antagonistic isolate was inoculated 1 cm from the edge of a petri plate containing Potato Dextrose Agar (PDA) medium. A 7-day-old mycelial disc (5 mm in diameter) of *M. oryzae* was placed on the opposite side of the PDA plate (9 cm in diameter). The PDA Petridish containing only *M. oryzae* was used as control. All plates were incubated at 28°C for 7 days. Following seven days of incubation period at 28°C, the diameter of the *M. oryzae* culture in the control and dual culture plates was measured in order to calculate the percentage of inhibition of radial growth (PIRG) using the formula below:


$$\%I=\frac{C-T}{C}\times 100$$

where I represent inhibition of mycelial growth, C represents fungal colony growth in the control plate, and T is the fungal colony growth in the dual culture plate.

### Biochemical characterization

Biochemical characterization including gram staining, catalase test, gelatin hydrolysis, citrate utilization test, starch hydrolysis, indole test, HCN test, casein hydrolysis and cellulase test was performed for the effective strains in our previous study (Mohapatra et al., 2023).

### Extraction of genomic DNA and genome sequencing

The genomic DNA of the most effective strain, *S. caeruleatus* was extracted using the Cetyl Trimethyl Ammonium Bromide (CTAB) (Hopwood, 2000). Extracted DNA was treated with RNase A to remove any RNA in the preparation and purified. Bacterial identification was conducted by 16S rRNA gene sequences through PCR using two universal primers, 27F (5′-AGAGTTTGATCCTGGCTCAG-3′) and 1492R (5′-GGTTACCTTGTTACGACTT-3′), followed by Sanger sequencing. The amplification was performed using an automated thermocycler (Takara) with the following conditions: an initial denaturation at 94°C for three minutes, followed by 40 cycles of denaturation at 94°C for thirty seconds, annealing at 50°C for one minute, and elongation at 72°C for ten minutes. DNA quality and quantity were assessed by gel electrophoresis and NanoDrop respectively. The identified 16S rRNA sequences were aligned with the National Center for Biotechnology Information (NCBI) database using BLAST. Sanger sequencing of the partial 16S rRNA gene allowed for genus-level identification before proceeding with whole-genome sequencing. For whole-genome sequencing (WGS), the PCR product of the bacterial DNA was sent to Novelgene Technologies Pvt. Ltd. in Hafeezpet, Hyderabad. WGS was conducted on the Illumina NovaSeq6000 (PE 150) following standard Illumina protocols.

### Genome assembly

The genome of the bacterial strain *S. caeruleatus* was comprehensively analyzed by submitting the sequencing reads to the Pathosystems Resource Integration Center (PATRIC) (Wattam et al., 2017). The sequencing data, containing adapter sequences and low-quality reads, was pre-processed using FASTP v0.23.4 to obtain high-quality reads, with a quality threshold of Q20. Reads with a mapping quality below Q20 were excluded from further analysis. The quality-trimmed reads were subsequently *de novo* assembled using SPAdes v3.15.5 with error correction (Bankevich et al., 2012). Scaffolding of the assembled contigs was performed using RagTag v2.1.0, employing a reference genome (GCF_001514235.1). The assembled genome’s contiguity and completeness were assessed using single-copy orthologs with QUASTv5.1 (Quality Assessment Tool for Genome Assemblies) (Gurevich et al., 2013) and BUSCO v5.7.0 (Benchmarking Universal Single-Copy Orthologs) (Simao et al., 2015). The BUSCO pipeline, run against the bacteria_odb10 database, was used for quantitative analysis of the assembly’s completeness.

### Genome annotation

The RASTtk v1.3.0 was used to annotate the genome of *S. caeruleatus* (Brettin et al., 2015) and genetic code 11. This process involved comparing the genome to others in PATRIC to identify gene functions, classify them into functional categories (Subsystems), and perform phylogenetic analysis. The Gene Ontology (GO) assignments, Enzyme Commission (EC) numbers, and KEGG pathways were determined using the blastKOALA server to annotate gene functions and pathways. The genome of *Streptomyces* was also annotated for genus-specific protein families (PLFams) and cross-genus protein families (PGFams) (Davis et al., 2016). Additionally, eggNOG-mapper v2.1.11 was used to identify clusters of orthologous groups (COG) for the predicted protein sequences. The complete genome was screened against known sequences in databases like CARD, NDARO, DrugBank, TTD, TCDB, PATRIC-VF, and Victors in order to identify antibiotic resistance genes, transporters, drug targets, and virulence factors. The Genome Annotation Service in PATRIC uses a k-mer-based method for detecting antimicrobial resistance (AMR) genes, using PATRIC’s curated collection of representative AMR gene sequences. A subsystem consists of a collection of proteins that collaborate to carry out a particular biological activity or create a structural complex. PATRIC annotation comprises the analysis of subsystems unique to each genome (Overbeek et al., 2005).

### Phylogenetic analysis

The phylogenetic tree was generated using the complete genome sequence of the *S. caeruleatus* S14, with additional whole genome sequences downloaded from the NCBI database. Using Molecular Evolutionary Genetics Analysis software (MEGA-XI), the Neighbor-Joining technique, aligned with MUSCLE, was used to deduce the evolutionary history (Saitou and Nei, 1987). The branches are accompanied with the percentage of replicate trees in which the linked taxa clustered together in the bootstrap test (1000 replicates) (Felsenstein, 1985). This analysis involved total 19 different strains of *Streptomyces* which included an outgroup *Pseudomonas aeruginosa* strain PPF-1. The genetic distance was analyzed using the Kimura 2-parameter model in MEGA software.

## Results

### Isolation and screening of *Streptomyces* spp.

**Figure 1:**
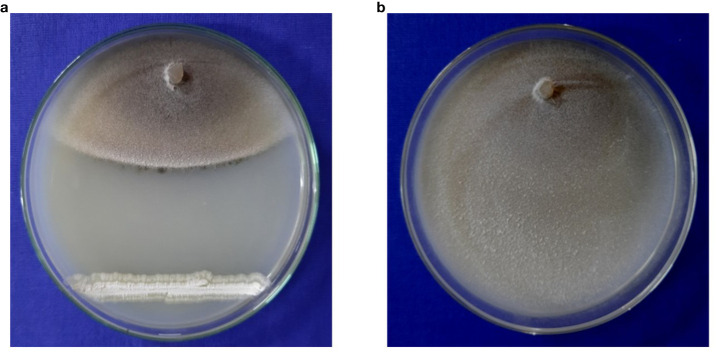
Antifungal activity of *Streptomyces caeruleatus* **(a)** Dual culture experiment of *streptomyces caeruleatus* against *Magnaporthe oryzae* **(b)** Isolate of *Magnaporthe oryzae* without biocontrol agent as control.

**Figure 2:**
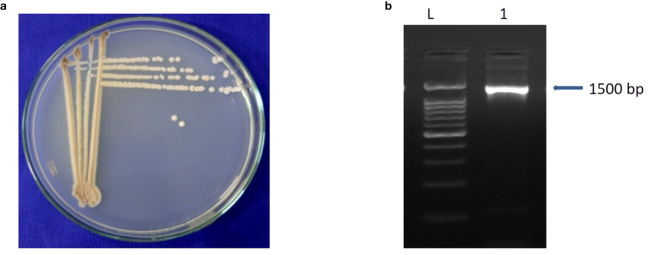
Characteristics of *Streptomyces caeruleatus* **(a)** colony morphology **(b)** PCR amplicons of 16S rRNA (L- 100 bps Ladder, 1- *Streptomyces caeruleatus*). Forward primer 27F (5’AGAGTTTGATCCTGGCTCAG-3’) and reverse primer 1492R (5’ GGTTACCTTGTTACGACTT-3).

A total of 85 isolates of *Streptomyces* spp. were isolated from different rice growing areas of Odisha, Eastern India. All the isolates were tested against *M. oryzae*. The result demonstrated that the *S. caeruleatus* S14 isolate exhibited a high level of inhibition, with an average inhibition area of 74.7% compared to other isolates. The untreated control displayed no inhibitory activity against *M. oryzae*, indicating the maximum growth of fungus (**Figure 1**; **Table 1**). PCR amplification of 16S rRNA gene showed an amplicon size of 1500bp (**Figure 2**).

**Table 1:** Screening of different *Streptomyces* spp. against *M. oryzae* for antifungal activity *in vitro.*.

Sl. No.	Isolate No.	Mean radial growth of pathogen (mm)	Mean percentage of inhibition	Sl. No.	Isolate No.	Mean radial growth of pathogen (mm)	Mean percentage of inhibition
1	S-1	58.50^bc^	35.00	44	S-44	71.07^d^	21.04
2	S-2	59.17^ab^	34.26	45	S-45	69.30^g-m^	23.00
3	S-3	32.90^h^	63.44	46	S-46	66.20^q-t^	26.44
4	S-4	70.17^d-i^	22.04	47	S-47	70.67^d-g^	21.48
5	S-5	57.37^c^	36.26	48	S-48	69.30^g-m^	23.00
6	S-6	58.73^abc^	34.74	49	S-49	71.10^d^	21.00
7	S-7	58.60^bc^	34.89	50	S-50	49.37^d^	45.15
8	S-8	60.07^za^	33.26	51	S-51	68.67^i-o^	23.70
9	S-9	70.90^def^	21.22	52	S-52	68.77^h-o^	23.59
10	S-10	58.13^bc^	35.41	53	S-53	67.97^k-p^	24.48
11	S-11	74.83^b^	16.85	54	S-54	68.20^j-p^	24.22
12	S-12	58.70^abc^	34.78	55	S-55	68.33^j-o^	24.07
13	S-13	67.87^l-p^	24.59	56	S-56	68.97^h-n^	23.37
14	S-14	22.77^i^	74.70	57	S-57	66.70^p-s^	25.89
15	S-15	67.57^n-q^	24.93	58	S-58	62.13^xy^	30.96
16	S-16	38.77^f^	56.93	59	S-59	41.77^e^	53.59
17	S-17	68.10^j-p^	24.33	60	S-60	68.77^h-o^	23.59
18	S-18	58.30^bc^	35.22	61	S-61	67.43^n-q^	25.07
19	S-19	70.30^d-h^	21.89	62	S-62	63.33^wx^	29.63
20	S-20	35.87^g^	60.15	63	S-63	63.50^vwx^	29.44
21	S-21	73.70^bc^	18.11	64	S-64	63.37^wx^	29.59
22	S-22	70.33^d-h^	21.85	65	S-65	61.03^yz^	32.19
23	S-23	68.93^h-n^	23.41	66	S-66	68.10^j-p^	24.33
24	S-24	69.53^e-k^	22.74	67	S-67	58.13^bc^	35.41
25	S-25	38.87^f^	56.82	68	S-68	67.73^m-p^	24.74
26	S-26	50.47^d^	43.93	69	S-69	68.67^i-o^	23.70
27	S-27	68.17^j-p^	24.26	70	S-70	62.23^xy^	30.85
28	S-28	69.37^g-l^	22.93	71	S-71	72.57^c^	19.37
29	S-29	69.40^f-l^	22.89	72	S-72	71.07^d^	21.04
30	S-30	68.80^h-n^	23.56	73	S-73	67.53^n-q^	24.96
31	S-31	69.63^d-j^	22.63	74	S-74	67.67^n-q^	24.82
32	S-32	68.03^k-p^	24.41	75	S-75	65.90^rst^	26.78
33	S-33	72.63^c^	19.30	76	S-76	62.83^wx^	30.19
34	S-34	70.93^de^	21.19	77	S-77	68.97^h-n^	23.37
35	S-35	57.97^bc^	35.59	78	S-78	69.63^d-j^	22.63
36	S-36	70.77^d-g^	21.37	79	S-79	63.90^uvw^	29.00
37	S-37	39.23^f^	56.41	80	S-80	64.83^tuv^	27.96
38	S-38	67.20^o-r^	25.33	81	S-81	62.27^xy^	30.82
39	S-39	65.433^st^	27.30	82	S-82	68.93^h-n^	23.41
40	S-40	39.633^f^	55.96	83	S-83	58.97^ab^	34.48
41	S-41	65.83^rst^	26.85	84	S-84	65.23^tu^	27.52
42	S-42	63.50^vwx^	29.44	85	S-85	68.37^j-o^	24.04
43	S-43	68.87^h-n^	23.48	86	Control	90.00^a^	0.00
					SE(m)	0.456	0.507
					CD@5%	1.274	1.415

Values represent the mean of three replicates. Means within a column sharing the same superscript letters are not significantly different, as determined by Duncan’s multiple range test (DMRT) at P ≤ 0.05.

### Comprehensive genome analysis

Whole genome sequencing analysis of the *S. caeruleatus* strain S14 revealed 186 contigs, with a total length of 9,750,804 bp (9.7 Mb). The N50 contig length, representing the shortest sequence length that accounts for 50% of the genome, is 603,168 bp. The genome has an average coverage of 119X and a G+C content of 71.03%. The L50 value, indicating the minimum number of contigs that together represent 50% of the genome’s length, is 7. Annotation statistics and comparisons with other genomes of the same species in PATRIC demonstrated that the genome quality is excellent, with a coarse consistency of 97.9%, fine consistency of 93.7%, and completeness of 99.9% (**Supplementary Table S2**). The *S. caeruleatus* S14 genome was annotated with the RAST toolkit (RASTtk), assigned the unique genome identifier 661399.5. The genome was annotated with genetic code 11 and categorized within the superkingdom Bacteria. This genome is classified as follows: cellular organisms > Bacteria > Terrabacteria group > Actinomycetota> Actinomycetes > Streptomycetales> Streptomycetaceae> Streptomyces >*Streptomyces caeruleatus*.

**Figure 3:**
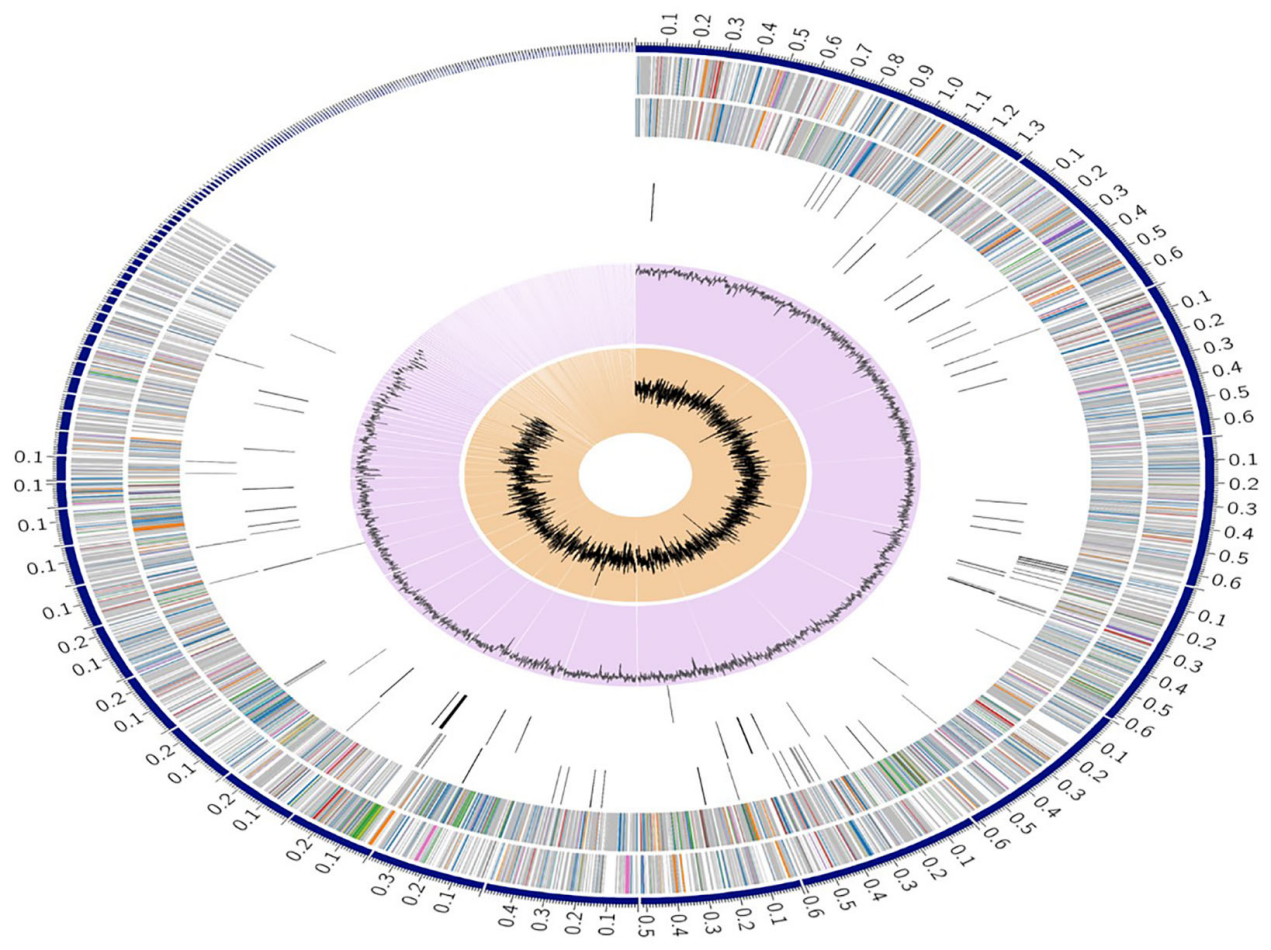
A circular graphical display of the distribution of the genome annotations. This includes, from outer to inner rings, the contigs, CDS on the forward strand, CDS on the reverse strand, RNA genes, CDS with homology to known antimicrobial resistance genes, CDS with homology to known virulence factors, GC content and GC skew. The colors of the CDS on the forward and reverse strand indicate the subsystem that these genes belong to.

The genome of *S.caeruleatus* strain S14 comprises 9,191 protein-coding sequences (CDS) (**Supplementary Table S3**), 68 transfer RNA (tRNA) genes, and 6 ribosomal RNA (rRNA) genes (**Figure 3**). Additionally, the genome contains 2,992 hypothetical proteins and 6,199 proteins with functional assignments. Among these, 1,562 proteins have Enzyme Commission (EC) numbers, 1,365 have Gene Ontology (GO) assignments, and 1,208 proteins are mapped with pathway assignments. The genome also includes 8,020 proteins belonging to genus-specific protein families (PLfams) and 8,199 proteins belonging to cross-genus protein families (PGFams) (**Supplementary Table S4**; **Table 2**).

**Table 2:** General genome features of *Streptomyces caeruleatus*.

Assembly Statistics features	Numbers/Values
Contigs	186
GC Content	71.03
Contigs L50	7
Genome Length	9,750,804 bp
Contig N50	603168
Coverage X	119
CDS	9,191
tRNA	68
rRNA	6
Hypothetical proteins	2,992
Proteins with functional assignments	6,199
Proteins with EC number assignments	1,562
Proteins with GO assignments	1,365
Proteins with Pathway assignments	1,208
Proteins with PATRIC genus-specific family (PLfam) assignments	8,020
Proteins with PATRIC cross-genus family (PGfam) assignments	8,199

**Figure 4:**
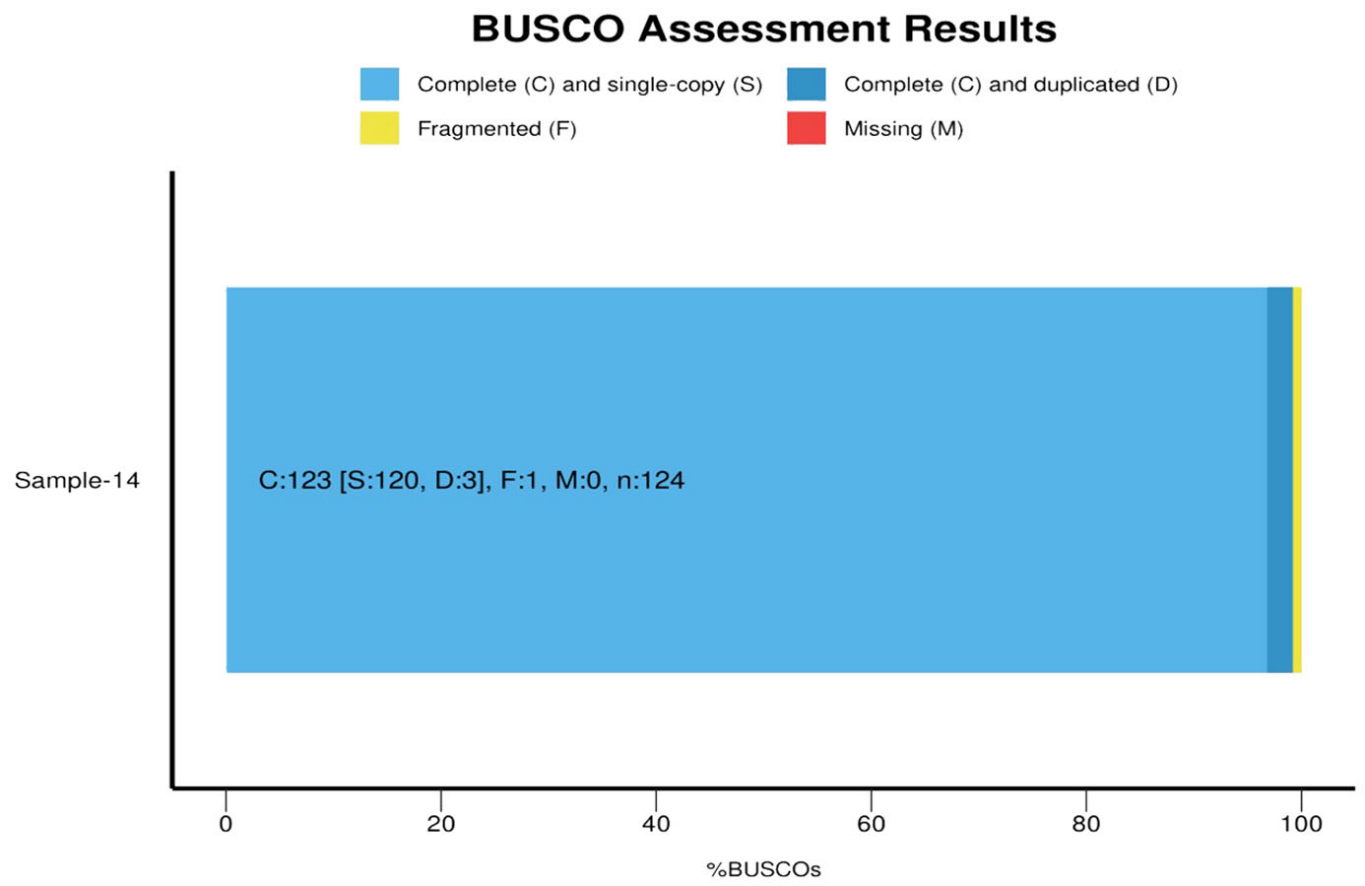
BUSCO analysis of assembled genome *Streptomyces caeruleatus*.

Quantitative analysis of the assembly’s completeness was performed using the BUSCO pipeline against the bacteria_odb10 database, identifying 99.2% of orthologous genes present, with only 1 gene fragmented. The BUSCO score was C:99.2% [S:96.8%, D:2.4%], F:0.8%, M:0.0%, n:124 (**Supplementary Table S5**; **Figure 4**). All of the protein-coding genes were allocated to clusters of orthologous groups (COGs) and classified into different functional categories using EggNOG mapper (**Supplementary Table S6**). In addition to these features, the genome annotation identified various biosynthetic gene clusters, which are crucial for the production of secondary metabolites. This highlights the potential of *S. caeruleatus* S14 for producing novel antibiotics and other bioactive compounds. The detailed analysis of regulatory elements and signaling pathways also provides insights into the complex regulatory networks governing the organism’s metabolic processes and environmental interactions. The comprehensive genomic characterization of *S. caeruleatus* S14 underscores its significance in biotechnology and its potential applications in agriculture, particularly in biocontrol and plant growth promotion.

The genome of *S.caeruleatus* includes specialty genes viz., antibiotic resistance-54 from (PATRIC), antibiotic resistance-6 (CARD), antibiotic resistance -2 (NDARO), drug target -6 (TTD), drug target-18 (Drug Bank), transporter-64 (TCDB), virulence factor -5 (Victors), and virulence factor -7 (Patric VF) (**Table 3**).

**Table 3:** Specialty genes in the genome of *Streptomyces caeruleatus*.

	Source	Genes
Antibiotic resistance	CARD	6
Antibiotic resistance	NDARO	2
Antibiotic resistance	PATRIC	54
Drug target	DrugBank	18
Drug target	TTD	6
Transporter	TCDB	64
Virulence factor	PATRIC_VF	7
Virulence factor	Victors	5

**Figure 5:**
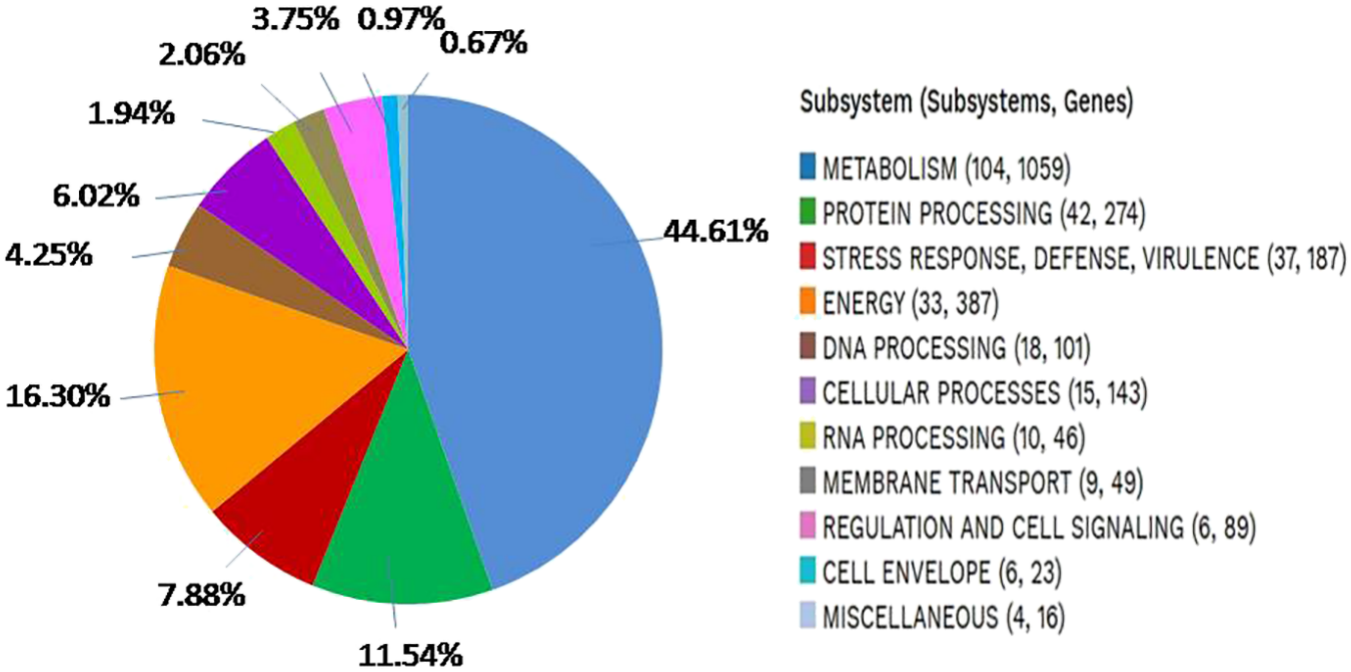
An overview of the subsystems of genome *Streptomyces caeruleatus*.

The analysis of *S. caeruleatus* for the presence of subsystems revealed the occurrence of superclasses, each containing varying numbers of subsystems (SS) and associated families/genes, as detailed in (**Figure 5**). The genome of *S. caeruleatus* contains several distinct superclasses, including: metabolism (104 SS, 1059 Genes), stress response, virulence, defense (37 SS, 187 Genes), protein processing (42 SS, 274 Genes), energy (33 SS, 387 Genes), DNA processing (18 SS, 101 Genes) cellular processes (15 SS, 143 Genes), RNA processing (10 SS, 46 Genes), membrane transport (9 SS, 49 Genes), regulation and cell signaling (6 SS, 89 Genes), cell envelope (6 SS, 23 Genes), and miscellaneous (4 SS, 16 Genes).

Groups of genes potentially contributing to the antimicrobial activity of bacterial strains were explored. The *S. caeruleatus* genome was annotated with various genes associated with different AMR mechanisms including those involved in efflux pump, genes conferring resistance, antibiotic target, enzymatic activation and inactivation and protein alteration. This comprehensive annotation offers insights into the potential antimicrobial resistance (AMR) mechanisms in *S. caeruleatus*, enhancing the understanding of its antimicrobial properties (**Table 4**).

**Table 4:** Antimicrobial resistance genes in the genome of *Streptomyces caeruleatus*.

AMR Mechanism	Genes
Antibiotic activation enzyme	KatG
Antibiotic inactivation enzyme	TEM family
Antibiotic target in susceptible species	Alr, Ddl, dxr, EF-G, EF-Tu, folA, Dfr, folP, gyrA, gyrB, inhA, fabI, Iso-tRNA, kasA, MurA, rho, rpoB, rpoC, S10p, S12p
Antibiotic target protection protein	Tet
Antibiotic target replacement protein	FabG, HtdX
Efflux pump conferring antibiotic resistance	Otr(C)
Gene conferring resistance via absence	gidB
Protein altering cell wall charge conferring antibiotic resistance	GdpD, MprF, PgsA
Protein altering cell wall structure conferring antibiotic resistance	VanH, VanJ, VanX
Regulator modulating expression of antibiotic resistance genes	LpqB, MtrA, MtrB, VanO-type

### Phylogenetic analysis

**Figure 6:**
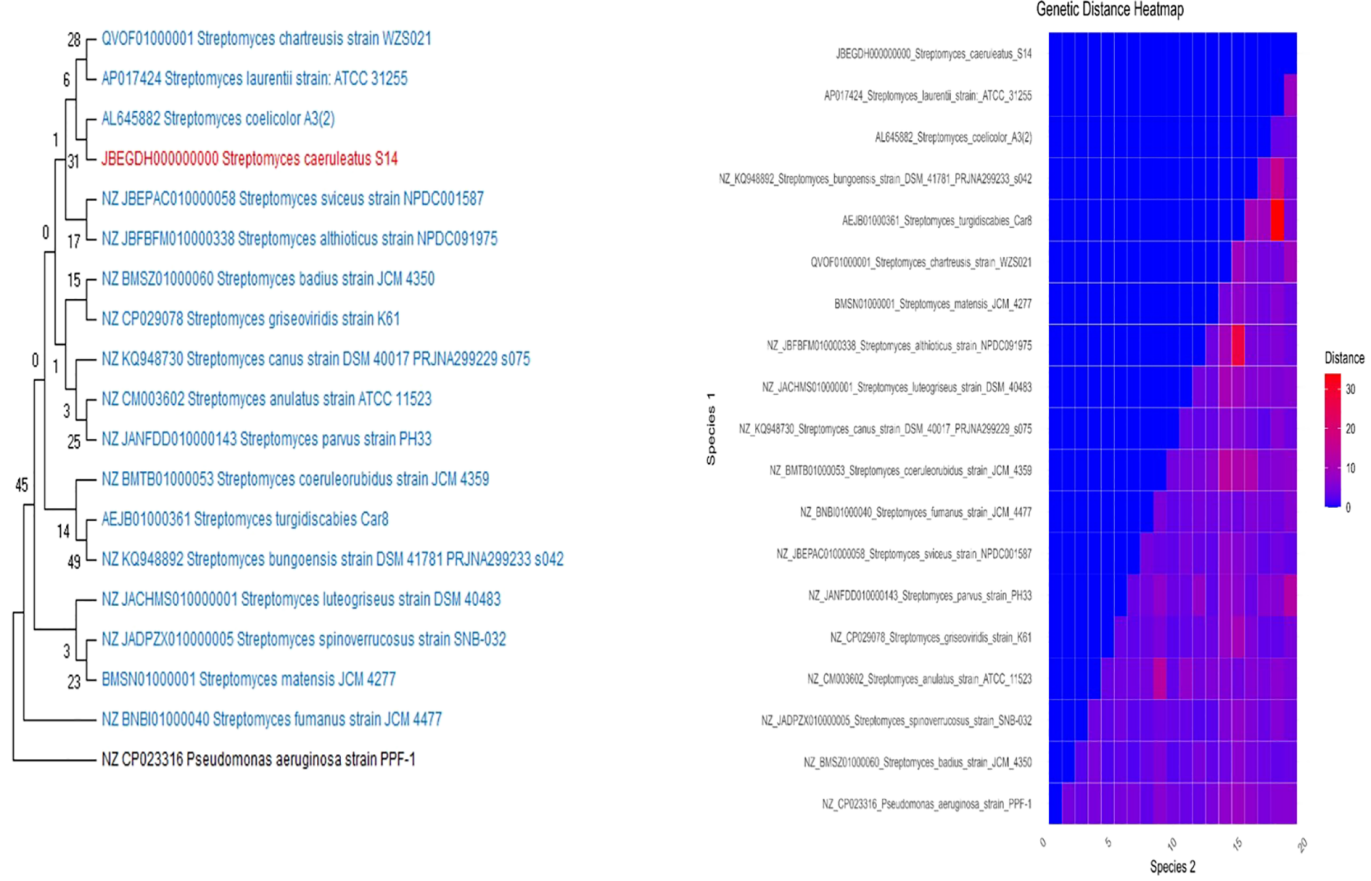
Phylogenetic analysis of the genome of *Streptomyces caeruleatus* S14. The neighbour joining tree was computed with Mega 11.0 with 1000 boot strap replications and a cut off value of 90%. The genetic distance heatmap was generated using Kimura 2 parameter model in MEGA.

Whole genome sequencing of the bacterial strain was identified as *S. caeruleatus* S14. Its evolutionary relationship was ascertained by constructing a phylogenetic tree with 1000 bootstrap replicates using the Neighbor-Joining method in the MEGA XI application, as illustrated in (**Figure 6**). According to the derived phylogenetic tree, *Streptomyces coelicolor*A3(2) and *S. caeruleatus* S14 have the closest genetic relationship, indicating a strong evolutionary connection between the two strains. Based on this analysis, it is proposed that *S. caeruleatus* S14 is a novel member of the *Streptomyces* genus.

## Discussion

Rice blast, caused by *M. oryzae*, is the most destructive disease affecting rice crops in rice-growing regions across the globe (Asibi et al., 2019). In sustainable agriculture, biocontrol agents offer an environmentally safe alternative to synthetic fungicides. This study offers a detailed genomic and functional analysis of the *S. caeruleatus* strain S14, isolated from the rice rhizosphere in Cuttack, Odisha and demonstrates significant biocontrol potential against *M. oryzae*, the pathogen responsible for rice blast disease, which affects rice crops worldwide (Law et al., 2017). The inhibition rate 74.7% recorded in dual culture assays makes *S. caeruleatus* as a highly effective biocontrol agent. This finding is particularly critical in the context of sustainable agriculture, where the reliance on chemical fungicides has led to environmental degradation, the evolution of fungicide-resistant strains, and concerns over food safety. Our findings are in concurrence with other workers who reported that *Streptomyces vinaceusdrappus*, isolated from Loktak lake sediment, inhibited the mycelial growth of *M. oryzae* by 53.5% (Ningthoujam et al., 2009). Similarly, *Streptomyces philanthi* RM-1-138, isolated from the chili pepper rhizosphere soil in southern Thailand, was found to inhibit the *in-vitro* growth of *M. oryzae* PTRRC-18 (Boukaew and Prasertsan, 2014). The mode of action of biological control agents are typically classified into four categories viz., the activity of substances, parasitism, competition, and antibiosis (Weller, 1988). However, the superior performance of *S. caeruleatus* in our study could be attributed to its unique genomic composition.

The complete genome analysis of *S. caeruleatus* S14 revealed a genome size of 9.7 Mb with 9191 protein-coding genes, including several associated with antimicrobial resistance, stress response, and virulence factors. These findings underscore the genetic basis for the strain’s biocontrol activity and provide insights into its potential to produce secondary metabolites, including antibiotics. Notably, genes encoding antibiotic resistance mechanisms, such as efflux pumps and antibiotic-inactivating enzymes, were identified, suggesting that *S. caeruleatus* could survive in competitive microbial environments. The high GC content (71.03%) of *S. caeruleatus* suggests an adaptation to soil environments, where high-GC content genomes are often correlated with the production of bioactive compounds. Notably, genome annotation identified several biosynthetic gene clusters (BGCs) responsible for the production of secondary metabolites, reinforcing the potential of strain for novel bioactive compound discovery. These BGCs are essential in the synthesis of antimicrobial compounds, which likely contribute to the observed inhibition of *M. oryzae*. A circular map of the genome was generated in GC View (Stothard and Wishart, 2005). The subsystems of genome were analyzed to gain insight into the precise role of a group of proteins responsible for a certain biological function or structural complex. Annotation entails examining the subsystems unique to each genome, which aligns well with the findings reported by (Settu et al., 2024). CDS from *S.caeruleatus* were analyzed and assigned to Clusters of Orthologous Groups (COG) by EggNOG (Huerta-Cepas et al., 2019).

Phylogenetic analysis confirmed the classification of the strain as a novel member of the genus *Streptomyces*. Its distinct genetic makeup compared to closely related species such as *S. coelicolor* further supports the uniqueness of this strain. This distinctiveness, combined with its biocontrol potential, makes *S. caeruleatus* as a viable option for subsequent exploration in sustainable agriculture practices. *Streptomyces* species carry antibiotic resistance genes, which contribute to their potential bioactive properties. Many *Streptomyces* species are known for producing secondary metabolites, including antibiotics. The genome of *S. caeruleatus* was annotated with genes homologous to known antibiotic-resistance genes. This resistance is considered as natural, consistently inherited trait specific to certain bacterial genera or species. The majority of shared genes are linked to various antimicrobial resistance mechanisms, such as antibiotic-target-modifying enzymes (RlmA(II)), antibiotic-inactivating enzymes (CatA family, FosB, Vgb(A)), replacement proteins (FabK, FabL), protection proteins (Lsa(B)), proteins involved in antibiotic sequestration (FabK-like), and regulators that modulate the expression of antibiotic resistance genes (BceR, BceS, LiaF, LiaR, LiaS) (Settu et al., 2024).

Given the promising results of this study, further research should focus on field trials to validate the efficacy of *S. caeruleatus* under natural environmental conditions. Additionally, studies on the formulation and delivery methods of this biocontrol agent will be crucial for its successful adoption in agriculture. The genetic data generated from this study also pave the way for exploring the metabolic pathways involved in secondary metabolite production, which could result in the discovery of novel compounds with agricultural and pharmaceutical applications.

## Conclusion

*S. caeruleatus* strain S14 is a potent biocontrol agent with significant promise for managing rice blast disease. Its comprehensive genomic profile, including antimicrobial resistance mechanisms, and stress response genes, highlights its adaptability and effectiveness. Biocontrol agents provide a sustainable alternative to chemical pesticides, contributing to both crop protection and environmental preservation. As global agricultural practices shift toward sustainability, the role of microbial agents like *S. caeruleatus* will become increasingly important in achieving food security and environmental health.

## Data Availability

The Genbank accession number for the 16S rRNA gene sequence is PP439819. The Genbank accession number for the whole genome sequence is JBEGDH000000000. The data were submitted in NCBI as name of SRA (Sequence Read Archieve) submission- (Accession No. SRR29262195, SRP511307). This whole-genome shotgun (WGS) project has been deposited at Gen-Bank under the accession BioProject- PRJNA1117618, BioSample- SAMN41577059.
