# Supplementary material for: Comprehensive genome analysis of Streptomyces caeruleatus S14 isolated from rice rhizosphere
Source: Front Plant Sci. 2025 Mar 26;16:1526700. doi: 10.3389/fpls.2025.1526700 (PMC11979203; doi:10.3389/fpls.2025.1526700)
Supplement: Supplementary file 1 [file Table1.doc]

Supplementary table 1: Collection of *Streptomyces* spp. from major rice growing areas of Odisha during 2022-23

| **Sl. No.** | **Isolate** Name | **District** | **Location** | **Coordinates** |
| --- | --- | --- | --- | --- |
| 1 | S-1 | Khordha | RRTTS Campus | 20.2648°N, 85.7985°E |
| 2 | S-2 |  | OUAT Campus | 20.2660°N, 85.8055°E |
| 3 | S-3 |  | Nirakarpur | 19.9987°N, 85.5393°E |
| 4 | S-4 |  | Jatani | 20.1713°N, 85.6990°E |
| 5 | S-5 |  | Kusumi | 19.9582°N, 85.4879°E |
| 6 | S-6 |  | Jankia | 20.0306°N, 85.5153°E |
| 7 | S-7 |  | Balipatana | 20.1931°N, 85.9621°E |
| 8 | S-8 | Cuttack | Biribati | 20.4242°N, 85.9632°E |
| 9 | S-9 |  | Tangi | 20.5642°N, 85.9973°E |
| 10 | S-10 |  | Athagarh | 20.5198°N, 85.6474°E |
| 11 | S-11 |  | Hajiipur | 20.3526°N, 86.0446°E |
| 12 | S-12 |  | Banki | 20.3975°N, 85.5781°E |
| 13 | S-13 |  | Kandarpur | 20.4651°N, 85.6528°E |
| 14 | S-14 |  | NRRI Campus | 20.45166°N, 85.9333°E |
| 15 | S-15 | Jagatsinghpur | Raghunathpur | 20.3287°N, 86.2336°E |
| 16 | S-16 |  | Balikuda | 20.2280°N, 86.1820°E |
| 17 | S-17 |  | Jhankada | 20.2845°N, 86.3014°E |
| 18 | S-18 |  | Biridi | 20.3554°N, 86.0508°E |
| 19 | S-19 |  | Kujanga | 20.3176°N, 86.5356°E |
| 20 | S-20 |  | Tirtol | 20.2976°N, 86.3411°E |
| 21 | S-21 |  | Ersama | 20.2010°N, 86.3991°E |
| 22 | S-22 |  | Naugaon | 20.1150°N, 86.1928°E |
| 23 | S-23 | Puri | Pipili | 20.1399°N, 85.8433°E |
| 24 | S-24 |  | Kakatpur | 19.9956°N, 86.1970°E |
| 25 | S-25 |  | Gop | 19.9922°N, 86.0222°E |
| 26 | S-26 |  | Brahmagiri | 19.7969°N, 85.6274°E |
| 27 | S-27 |  | Nimapara | 20.0762°N, 85.9929°E |
| 28 | S-28 |  | Delang | 20.0396°N, 85.7678°E |
| 29 | S-29 |  | Astarang | 19.9803°N, 86.2656°E |
| 30 | S-30 | Kendrapara | Indupur | 20.6079°N, 86.4031°E |
| 31 | S-31 |  | Rajkanika | 20.7308°N, 86.7108°E |
| 32 | S-32 |  | Chandol | 20.4868°N, 86.2929°E |
| 33 | S-33 |  | Baro | 20.5481°N, 86.4576°E |
| 34 | S-34 |  | Derabish | 20.4922°N, 86.2895°E |
| 35 | S-35 |  | Pattamundai | 20.5754°N, 86.5749°E |
| 36 | S-36 | Bhadrak | Charampa | 21.0973°N, 86.5340°E |
| 37 | S-37 |  | Chandabali | 20.7981°N, 86.7579°E |
| 38 | S-38 |  | Kalimegha | 21.00491°N, 86.6336°E |
| 39 | S-39 |  | Mukundpur | 20.9964°N, 86.6230°E |
| 40 | S-40 |  | Basudevpur | 21.1439°N, 86.7405°E |
| 41 | S-41 |  | Arasa | 21.0813°N, 86.5500°E |
| 42 | S-42 |  | Dolasahi | 21.0346°N, 86.5940°E |
| 43 | S-43 |  | Nandapur | 21.0213°N, 86.6198°E |
| 44 | S-44 |  | Chabishpada | 20.9646°N, 86.6114°E |
| 45 | S-45 |  | Tihidi Purunahat | 20.9948°N, 86.6287°E |
| 46 | S-46 | Balasore | Soro | 21.2936°N, 86.6977°E |
| 47 | S-47 |  | Khirkona | 21.1992°N, 86.6980°E |
| 48 | S-48 |  | Markona | 21.1863°N, 86.6097°E |
| 49 | S-49 |  | Remuna | 21.5269°N, 86.8634°E |
| 50 | S-50 |  | Khantapada | 21.3848°N, 86.8376°E |
| 51 | S-51 |  | Bahanaga | 21.3434°N, 86.7634°E |
| 52 | S-52 |  | Simulia | 21.1782°N, 86.5967°E |
| 53 | S-53 | Bolangir | Loisinga | 20.8574°N, 83.5151°E |
| 54 | S-54 |  | Sibtala | 20.6680°N, 83.3219°E |
| 55 | S-55 |  | Belpara | 20.5921°N, 82.9700°E |
| 56 | S-56 |  | Bongamunda | 20.3394°N, 82.9074°E |
| 57 | S-57 |  | Tushura | 20.5037°N, 83.4844°E |
| 58 | S-58 |  | Agalpur | 21.0026°N, 83.4332°E |
| 59 | S-59 | Bargarh | Attabira | 21.3815°N, 83.7949°E |
| 60 | S-60 |  | Katapali | 21.4172°N, 83.6156°E |
| 61 | S-61 |  | Bhatli | 21.4714°N, 83.5355°E |
| 62 | S-62 |  | Barpali | 21.1851°N, 83.6050°E |
| 63 | S-63 |  | Padampur | 21.0108°N, 83.0956°E |
| 64 | S-64 | Ganjam | Bhanjanagar | 19.9359°N, 84.5897°E |
| 65 | S-65 |  | Chatrapur | 19.3709°N, 85.0089°E |
| 66 | S-66 |  | Hinjili Cut | 19.4750°N, 84.7474°E |
| 67 | S-67 |  | Chikiti | 19.2109°N, 84.5978°E |
| 68 | S-68 |  | Patrapur | 19.1339°N, 84.5755°E |
| 69 | S-69 | Jajpur | Binjharpur | 20.7343°N, 86.4037°E |
| 70 | S-70 |  | Barabati | 20.8010°N, 86.1535°E |
| 71 | S-71 |  | Brahmabarada | 20.7378°N, 86.2428°E |
| 72 | S-72 |  | Ramachandrapur | 20.6837°N, 86.3599°E |
| 73 | S-73 |  | Badachana | 20.6742°N, 86.1291°E |
| 74 | S-74 |  | Panikoili | 20.9132°N, 86.2344°E |
| 75 | S-75 |  | Korei | 20.9611°N, 86.1829°E |
| 76 | S-76 |  | Gurudaspur | 20.8622°N, 86.3041°E |
| 77 | S-77 |  | Ankula | 20.8551°N, 86.3088°E |
| 78 | S-78 | Nayagarh | Odagaon | 20.0204°N, 84.9880°E |
| 79 | S-79 |  | Balarampur | 20.0670°N, 85.3466°E |
| 80 | S-80 |  | Kendupalli | 20.2965°N, 85.2693°E |
| 81 | S-81 |  | Bindhyagiri | 20.4126°N, 85.0295°E |
| 82 | S-82 |  | Daspalla | 20.3394°N, 84.8532°E |
| 83 | S-83 |  | Bhapur | 20.2467°N, 85.2567°E |
| 84 | S-84 |  | Khandapada | 20.2687°N, 85.1811°E |
| 85 | S-85 |  | Fategarh | 20.3029°N, 85.3195°E |
